# Subcutaneous House Dust Mite Immunotherapy Effectiveness and Safety in a Paediatric Population: A Prospective Real-Life Study †

**DOI:** 10.3390/jcm14124188

**Published:** 2025-06-12

**Authors:** Inmaculada Buendía Jiménez, María Matas Ros, Teresa Garriga-Baraut, María Araceli Caballero-Rabasco, Amalui Vásquez Pérez, Laura Valdesoiro-Navarrete, Magdalena Lluch Pérez, Jesús Villoria, Alfons Malet i Casajuana

**Affiliations:** 1 Medical Department, Probelte Pharma, S.L.U., 30100 Murcia, Spain; mariamatas@probeltepharma.es; 2 Hospital Universitario Vall d’Hebrón, 08035 Barcelona, Spain; teresagarriga@hotmail.com; 3 Growth and Development Research Group, Vall d’Hebron Research Institute, 08035 Barcelona, Spain; 4 Hospital del Mar, 08003 Barcelona, Spain; macaballerorabasco@hmar.cat; 5 Department of Medicine and Life Sciences, Pompeu Fabra University, 08002 Barcelona, Spain; 6 Hospital Universitario Joan XXIII, 43005 Tarragona, Spain; avasquez.hj23.ics@gencat.cat; 7 Hospital Xarxa Santa Tecla, 43003 Tarragona, Spain; 8 Pediatric Nutrition and Human Development Research Unit, Rovira i Virgili University, 43002 Reus, Spain; 9 Hospital Universitario Parc Taulí, 08208 Sabadell, Spain; lvaldesoiro@gmail.com; 10 Parc Taulí Hospital Universitari, I3PT CERCA, 08208 Sabadell, Spain; 11 Centro Médico Teknon, 08022 Barcelona, Spain; 17585mlp@comb.cat (M.L.P.); 10655amc@comb.cat (A.M.i.C.); 12 Department of Design and Biometrics, Medicxact, 28430 Alpedrete, Spain; villoriajesus@medicxact.es

**Keywords:** allergic rhinitis, vaccination, immunologic desensitization, paediatrics, cohort studies, immunity

## Abstract

**Background/Objectives:** Allergen immunotherapy is the sole therapeutic option capable of modifying the natural course of allergic rhinitis and preventing the development of asthma. Results from paediatric patients are scarce. To evaluate the effectiveness and safety of a glutaraldehyde-modified extract of mites (Beltavac^®^) administered for one year under clinical routine conditions in children between 3 and 11 years old. **Methods:** This was a multicentre, prospective, 13-month cohort study. Among 97 children diagnosed with immunoglobulin E-mediated house dust mite allergic rhinoconjunctivitis, 87 initiated the subcutaneous immunotherapy. The main outcomes included the Combined Symptoms and Medication Score (CSMS), assessed for 1 month at baseline and after 1, 6, and 12 months, and the number of adverse reactions according to the WAO adverse reaction grading system. The levels of serum-specific immunoglobulins were also assessed. **Results:** CSMS improved scores throughout therapy (adjusted mean change and 95% confidence interval: 0.55, 0.26–0.84 points; *p* < 0.001). Improvements occurred in both children with (n = 68) and without asthma (n = 19), as well as in children aged ≥6 years (n = 76) and <6 years (n = 11), although statistical significance was not reached in the smallest subgroups. Eight children (9.2%) developed a total of 15 adverse reactions. Most occurred after the initial dose (five out of eight children), and were local (six out of eight) and minor (five out of eight). Over 90% of patients completed the full regimen. **Conclusions:** This study supports the effectiveness and safety of allergen immunotherapy administered according to a rush schedule for one year for paediatric allergic rhinitis.

## 1. Introduction

Allergic rhinoconjunctivitis (AR) is an immunoglobulin (Ig) E-mediated inflammatory disorder of the nasopharynx that occurs in response to typically innocuous environmental components [1,2]. It is common in children, with prevalences around 13–20% [1,3,4,5]. Nearly half of affected patients become symptomatic by the age of 6, although the prevalence increases with age [3]. Thus, the disease may already be present from early childhood, although symptoms may arise later [1]. In addition to the bothersome and well-known symptoms of sneezing, rhinorrhoea, and nasal obstruction, it also associates with facial or nasal deformity; ocular, nasal, or oral mucosal alterations [1]; disruptions to everyday life [2,6], including school performance [7,8]; and co-morbidities, among which asthma stands out [2]. When left untreated or treated inadequately, it can continue into adulthood [9].

A tiered approach to paediatric AR therapy is usually considered, involving exposure avoidance of triggering allergens, symptomatic pharmacotherapy, and disease-modifying immunotherapy [5]. Since effective exposure avoidance is usually difficult to achieve and side effects can limit the benefits of pharmacotherapy, immunotherapy turns out to be of particular interest in this setting [1,5]. In fact, it has the potential to speed up the maturation of the infant immune system, characterized by a hyperreactivity milieu, which could be surmounted by controlled exposure to microbial products [10,11,12,13,14]. This has led to postulating greater effectiveness in children than in adults, and there is evidence suggesting that immunotherapy may impede progression from AR to asthma and sensitization to new allergens [15,16]. However, it is not devoid of drawbacks, including a paradoxical allergenicity potential [15]. Studies of its benefits and risks in patients below 5 years of age are long needed [5,16,17]. Only a few studies have been performed that suggest subcutaneous immunotherapy to be safe and effective in preschool children with respiratory allergic diseases [18].

A number of innovations, including the purification and standardization of allergenic components and the incorporation of adjuvants and allergoids, have brought a number of improvements in terms of convenience, efficacy, and tolerability [19]. On the other hand, the high specificity of such components and the variety of factors that may affect the performance of finished products represent a challenge in terms of documented effectiveness [20]. Hence, a product-specific approach for the evaluation and reporting of results is important [17]. The level of available evidence supporting immunotherapy for perennial AR in children is lower than for adults and for seasonal conditions, and virtually restricted to sublingual preparations [5]. The Beltavac^®^ allergen immunotherapy is a glutaraldehyde-polymerized allergenic extract of house dust mites (HDMs) *D. pteronyssinus* and *D. farinae*, the most important species of a set of common indoor allergenic sources [2,21]. Prior retrospective studies in children with AR using subcutaneous rush regimes support the safety of this immunotherapy [22,23,24]. The present real-life one-year prospective cohort study aimed to expand the available evidence by assessing the safety and effectiveness of this subcutaneous Beltavac^®^ immunotherapy with polymerized HDM extract in children as young as 3 years old with persistent AR, with or without asthma. Recommended standardized clinical outcomes [25,26] were used in the evaluation, and the serum levels of specific relevant Ig were measured before and after completing the study.

## 2. Materials and Methods

### 2.1. Study Design

This was a non-interventional, multicentre, prospective cohort study. The study treatment was administered as part of the standard clinical practice. The immunotherapy follow-up lasted 12 months, a period long enough to assess the short-term effectiveness and safety of this kind of treatment [27].

All legal representatives signed their written informed consent before inclusion. The study protocol was approved by the Teknon Medical Centre Ethics Committee, Barcelona, Spain. Probelte Pharma S.L.U. provided funds for logistical aspects. The study complied with the protocols and principles of the Declaration of Helsinki, as well as the Good Clinical Practice (GCP) guidelines, and all other applicable regulations. Statistical analyses were performed by Medicxact CRO, Madrid, Spain. The study has been registered in the clinicaltrials.gov database with the code NCT03963947.

### 2.2. Study Population

The study population consisted of paediatric patients (aged 3–11 years) with active clinical manifestations of rhinitis/rhinoconjunctivitis associated or not with asthma, a positive skin prick test (mean papule diameter ≥ 3 mm) to a standardized allergenic extract of *D. pteronyssinus* and/or *D. farinae*, and a positive serum (class ≥ 3) allergen extract-specific IgE test.

Patients with a positive serum IgE test for other allergenic extracts that could interfere with the study results, those previously treated with the study-specific allergenic extract or other cross-reactive extracts, those with severe/uncontrolled asthma, autoimmune conditions, malignant neoplasms, congenital or acquired immunodeficiencies, cardiovascular diseases, or other chronic diseases (e.g., chronic infections, mental disorders), and those under treatment with β-blockers were excluded.

### 2.3. Exposure of Interest

The exposure of interest was the allergen immunotherapy Beltavac^®^. This is a polymerized extract of house dust mite mixture (ATC code: V01AA03), indicated in the hyposensitizing treatment of IgE-mediated allergic diseases. The allergenic extract is biologically standardized and polymerized for specific immunotherapy that is administered subcutaneously, with a prolonged release mechanism. The active ingredient is a mixture of 2 polymerized mites—*Dermatophagoides pteronyssinus* and *Dermatophagoides farinae* (1:1)—prepared at concentrations of 2 RC/mL (Der p1 = 15 µg/mL, Der p2 = 12 µg/mL, Der f1 = 22 μg/mL, Der f2 = 17 μg/mL, Der p 23 = 0.4 μg/mL). In addition to the allergen extract, the other components of the immunotherapy are aluminium hydroxide gel (adjuvant), sodium chloride, phenol (preservative), and water for injection.

#### 2.3.1. Study Procedures

**Figure 1:**
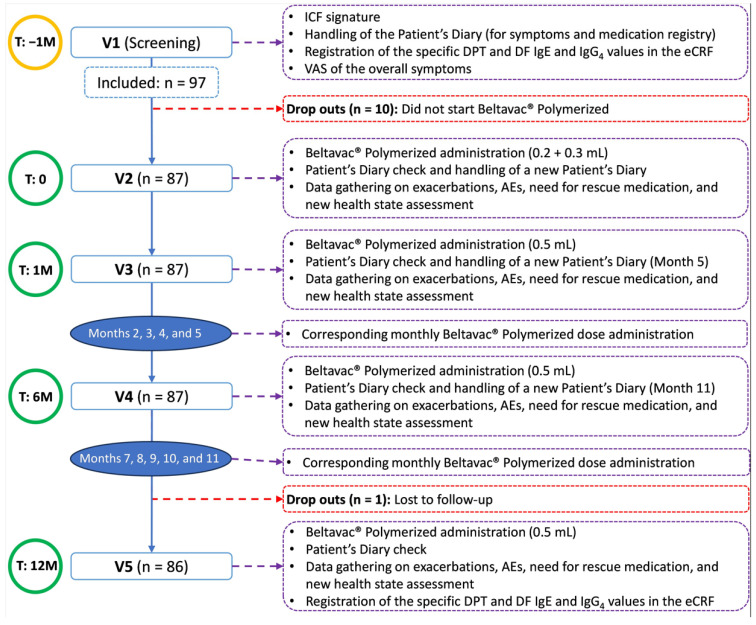
Flowchart. T: timepoint; V: visit; ICF: informed consent form; DPT: *Dermatophagoides pteronyssinus*; DF: *Dermatophagoides farinae*; Ig: immunoglobulin; eCRF: Electronic Case Report Form; VAS: visual analogue scale; AE: adverse event.

The investigators met with the parents or legal representatives of potential participants in order to inform them about all aspects of the study relevant to their decision. Consent was documented by a written, signed, and dated informed consent form, completed by at least one parent or legal representative. Study participants had five study-specific visits arranged over 13 months, approximately (Figure 1). Additional monthly visits for receiving immunotherapy doses could be either performed at the same study sites or shared with patients’ Primary Care facilities. Screening procedures, including the determination of serum IgE and IgG4 antibodies against the specific *D. pteronyssinus* and *D. farinae* allergens contained in the study immunotherapy and against the Der p 1, p 2, p 10, and p 23 antigens, and the checking of selection criteria, occurred at Visit 1. Treatment with Beltavac^®^ immunotherapy with polymerized HDM extract was started one month later (Visit 2), with the administration of 0.2 mL on one arm and 0.3 mL on the other, 30 min later. Subsequent doses took place at approximate monthly intervals, at doses of 0.5 mL in one of the arms. The first follow-up visit (Visit 3) coincided with the first full 0.5 mL dose. The second follow-up visit (Visit 4) occurred approximately six months after Visit 2. At Visit 5, 12 months after Visit 2, serum samples for the determination of specific Ig were taken again. For the collection of some outcomes (see below), patient diaries were used. These had to be completed by the patients themselves or their parents during the month preceding Visits 2 to 5.

#### 2.3.2. Outcomes

Following the European Academy of Allergy and Clinical Immunology (EAACI) position paper [24], the main outcome to assess the effectiveness of the immunotherapy was the recommended combined symptoms and daily medication score (CSMS). It featured the six rhinoconjunctivitis symptoms (CSMS6), yet a partial sub-score based on the four rhinitis symptoms (CSMS4) was also calculated, since these are particularly prominent in patients with mite allergies. It was assessed for 1 month at baseline and after 1, 6, and 12 months of immunotherapy. Based on these scores, the proportion of days without symptoms was also calculated. Patients also completed a 10 cm visual analogue scale (VAS) on their perceived health status related to allergy symptoms (0 = the worst possible condition; 10 = the best possible condition) on all study-specific visits. Safety outcome measures were the number of adverse events (AEs), coded according to the MedDRA’s terminology system, and severe AEs (SAE), and the number of adverse reactions, classified according to the World Allergy Organization’s grading system [26].

For patients with asthma, an analogous combined scale (ASMS) was used to assess daily the severity of allergic asthma symptoms and medication requirements. This scale was based on the Spanish Guidelines for Asthma Management (GEMA) version 4.3 [28], and used the same 0–3 scoring system as the rhinoconjunctivitis CSMS to yield a total score ranging from 0 to 6 (the highest to the worst). ASMS symptoms were scored as follows: 0 = no symptoms; 1 = diurnal symptoms (difficulty breathing, chest tightness, wheezing or cough) only; 2 = nocturnal symptoms; and 3 = limitation of physical activity. The medication intake was scored as follows: 1 = short-acting-β2 intake; 2 = inhaled corticosteroid low dose; 3 = inhaled corticosteroid moderate dose. In addition, the number of asthma exacerbations in the year preceding Visit 2 and throughout the study was collected.

Finally, serum-specific *Dermatophagoides pteronyssinus*, *Dermatophagoides farinae*, Der p1, Der p2, Der p10, Der p23, IgE, and IgG4 were assessed at baseline and after 1 year of allergen immunotherapy, when possible, in clinical practice.

#### 2.3.3. Statistical Analysis

Appropriate descriptive analyses were performed for all gathered data, including means, standard deviation (SD), quartiles, minimum and maximum values for continuous variables, and absolute and relative frequencies for categorical variables. Additionally, asymptotic confidence intervals (CIs) were calculated to estimate the unknown population parameters without correction for finite sampling, since their coverage is more conservative [29]. Symptom and medication scores were analysed within the generalized linear mixed-effects model framework over an intention-to-treat (ITT) analysis population. Note that these models can accommodate missing data whenever they occur at random (i.e., unrelated to disease severity). The presence of asthma was introduced by default as an explanatory variable in all models. However, final models of CSMS only included categorized age (≥6 years/<6 years) and the presence of conjunctivitis symptoms because the presence of asthma was not significantly associated with differences in outcomes. The incidence of asthma exacerbations was analysed using similar models as for CSMS, but using the Poisson distribution for errors and the observed exposure times as an offset to yield monthly incidences. Statistical analyses were performed with SAS software, version 9.4 (Cary, NC, USA).

The sample size was calculated as 92 patients, considering a finite population of about 450 paediatric patients with a diagnosis of permanent allergic rhinitis in Spain, to provide a precision (half-width) of 3% in a 90% bilateral confidence interval when the actual proportion of adverse reactions is as low as 4%, as in one of the aforementioned previous studies [29].

## 3. Results

### 3.1. Baseline Characteristics

Between July 2019 and June 2021, 97 patients were included; however, 10 patients withdrew before receiving any dose of Beltavac^®^ allergen immunotherapy. Of the remaining 87 patients, males accounted for 57 (65.5%) subjects, and the mean (SD) age was 8.2 (2.3) years; 76 patients (87.4%) were ≥6 years old. The mean (SD) body mass index was 17.9 (3.4) kg/m2. Almost all patients had rhinitis symptoms (n = 85; 97.7%), 59 (67.8%) had conjunctivitis, and 68 (78.2%) had asthma. These characteristics were comparable between patients with and without asthma (Table 1). Remarkably, 86 patients (98.9%) completed the 12-month follow-up, and 81 (93.1%) received a complete immunotherapy regimen.

Thirty-seven (42.5%) only showed a positive skin prick test to either or both standardized allergenic extracts of *D. pteronyssinus* and *D. farinae*, whilst the remaining fifty (57.5%) showed positivity to other allergens as well. Fifteen patients (17.2%) had at least one other concomitant immunological condition, with atopic dermatitis (12 patients, 13.8%) being the most common. Baseline outcomes scores (CSMS and ASMS) were indicative of mild symptoms in general; yet these were present for about two-thirds of the days on average, and the reported health status scores related to allergies were somewhat poor (Table 1).

### 3.2. Efficacy

**Figure 2:**
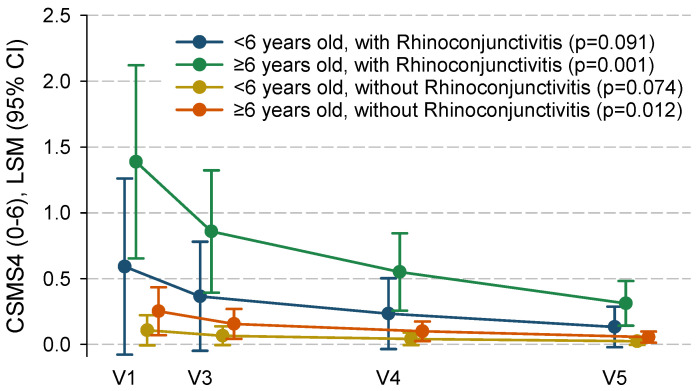
CSMS4 evolution throughout the study visits across different subgroups of subjects (obtained from the regression model). CSMS4: combined (rhinitis) symptoms and medication score based only on the four nasal daily symptoms score and daily medication score; 95% CI: 95% confidence interval; LSM: least square means.

There were progressive and significant CSMS6 reductions (of about 10 percent of the full-scale breadth) that occurred from baseline to each of the subsequent follow-up visits (Table 2). Reductions occurred in all individual symptoms (Table S1) and were particularly prominent in the symptoms sub-score among children without asthma, although statistical significance was not reached in the former subgroup, probably due to its small size (Table 2). Reductions were also significantly greater among children with conjunctivitis symptoms than in those showing only rhinitis symptoms, and numerically (not significantly) greater among children aged ≥6 years than in those aged under 6 years (Table 2). Reductions in the latter sub-group did not reach statistical significance, again probably because of insufficient statistical power due to its small size. The results for the CSMS4 sub-score were quite similar (Table 2, Figure 2). There was also a significant reduction in the proportion of symptom-free days that was comparable between patients with and without asthma, although statistical significance was not reached within these smaller sub-groups. VAS scores about perceived health status improved significantly in all sub-groups (Table 2).

Among children with asthma, the ASMS scores showed a marginally significant improvement (reduction), mainly attributable to asthma specific symptoms, but not medication (Table 2). There was also a significant reduction in the risk of asthma exacerbations with respect to the year preceding the study.

There were increases in serum-specific immunoglobulin levels. Numerically, these were greater for IgG4 than for IgE; statistical significance was reached for IgG4 anti-specific *D. pteronyssinus* and *D. farinae* allergens (Table S2, Figures S1 and S2).

### 3.3. Safety

Beltavac^®^ Polymerized was in general well tolerated. As mentioned, 81 out of the 87 patients (93.1%) completed the full treatment regimen. In total, 23 patients (26.4%) experienced AEs, which were serious in only 3 (3.5%) (Table 3). The most common AEs were infections and infestations, mostly upper respiratory tract infections, impacting 10 patients (11.5%) (all asthmatics). Respiratory, thoracic, and mediastinal disorders was the second most common system organ class affected, arising in eight patients (9.2%) (six asthmatics), with asthma exacerbations (n = 3) being the most common. The three serious AEs were one acute appendicitis episode that required appendicectomy and two severe asthma exacerbations requiring hospitalization, one before starting Beltavac^®^ therapy. None of them were related to the immunotherapy.

Additionally, eight patients (9.2%) experienced a total of 15 adverse reactions, none of which were considered severe. Only two were systemic: one grade 1 and another grade 2 (mild asthma exacerbation that required salbutamol administration). Treatment was required in six patients (6.9%), mostly oral antihistamines, although topical corticosteroids were required in two patients to treat localized erythema and wheals. These mostly occurred after the initial Beltavac^®^ doses (Table 3). Accounting for all doses administered, the rate of adverse reactions was 0.01 reactions per dose. The most common adverse reactions were classified as general disorders and administration site conditions (n = 8; 9.2%), with oedema in the administration site being the most common (n = 3; 3.5%).

## 4. Discussion

The present observational prospective cohort study shows a consistent association between the administration of Beltavac^®^ allergen immunotherapy with polymerized HDM extract under a convenient subcutaneous once-a-month rush protocol and clinical improvements during the first year of immunotherapy in children diagnosed with IgE-mediated allergic rhinoconjunctivitis, with and without asthma, sensitized to HDM allergens. Improvements are evident in age groups both below and above 6 years, both for rhinoconjunctivitis and asthma, in terms of symptoms and rescue medication requirements. Immunotherapy is well tolerated: over 90% of patients received the full allergen immunotherapy regimen and less than a handful experienced adverse reactions, with none of them being severe or serious.

The progressively longer time spent in well-insulated homes or office buildings has been associated with an ever-growing exposure to HDMs [30,31,32,33]. This, coupled with a reduced exposure to microbial products, preventing maturation of the infant immune system [10,11], can boost repeated infections, as well as autoimmune and allergic disorders, which are on the rise in developed countries [31,34,35]. Since completely eliminating environmental triggers is unfeasible, immunotherapy rises as the sole disease-modifying treatment currently available [25], preventing progression to asthma and lowering medication use [19]. Our findings of a progressive reduction in all assessed symptoms and medication regardless of age and the presence of asthma are consistent with those from similar studies [22,23,24,30,36,37] and randomized clinical trials in children and adolescents treated with specific HDM sublingual immunotherapy [38,39]. This study also provides support to the EAACI recommendation for immunotherapy in children with controlled HDM-driven allergic asthma as an add-on to regular asthma therapy [40], which is mostly based on adult data, suggesting that it might be extended to children as well. More generally, our results expand the evidence for a particular product, which is welcomed by the EAACI guidelines [5], and indicate that high-grade evidence supporting immunotherapy for paediatric HDM allergic rhinitis could be obtained with the tested immunotherapy in well-designed clinical trials.

Very succinctly, relevant immunologic mechanisms of tolerance induced by allergen immunotherapy include the modulation of dendritic cells’ response, which is associated with a shifting in T-cell differentiation. This fosters the expression of regulatory TH1 phenotypes at the expense of pro-allergic TH2 phenotypes. B-cell class switching also ensues, which can be traced by a transient upsurge in serum concentrations of specific IgE antibodies that subsides in about 3 to 6 months, and is followed by an enduring increase in specific IgG1, IgG4, and IgA inhibitory antibodies [41,42]. However, in long-term AIT studies, the level of specific IgE has been shown to decrease over time [43,44]. On the other hand, IgG values maintain the specific increases during the treatment period, but a decline has been reported after AIT termination [45]. Consequently, these trends seem to depend mainly on the duration of the study or the time of sampling and reflect immunogenicity and allergen exposure. Longer controlled clinical trials with serial determinations are necessary to further investigate their clinical relevance [46]. Since we measured Ig levels at the beginning of the study and one year later, we were only able to detect consistent (and significant) increases in specific IgG4. In turn, IgE values showed considerable variability. This suits the aforementioned mechanisms well, because a greater variability in IgE levels might reflect its transient nature, whilst IgG4 was more homogeneous. Similar patterns have been described in a previous study with sublingual immunotherapy in HDM-allergic children [38]. Consequently, the results suggest that Beltavac^®^ may achieve allergen tolerance (therapeutic effect), as stated in the EAACI guidelines on allergen immunotherapy for rhinoconjunctivatis after a minimum of 3 years of treatment [5,15,41]. The compliance of the patients to the monthly injections over the one-year follow-up was very high during the study—close to 100%. In the literature, the range of reported adherence varies from 18% to 90%, being higher in clinical studies than in real-life surveys [5], which is consistent with our results.

Lastly, subcutaneous Beltavac^®^ immunotherapy with polymerized HDM extract is well tolerated, presenting few adverse reactions, none of which are deemed severe. Cases of severe adverse reactions, including anaphylaxis, have been reported with the use of subcutaneous immunotherapy in children [15,47], but these were not observed in this study. Known drawbacks of subcutaneous immunotherapy include the discomfort from repeated injections, the dependence on a healthcare unit, long periods required for tolerance establishment, and the risk of severe skin hypersensitivity reactions. However, the improved safety of polymerized extracts allowed the development of the accelerated dosing regimen (rush regimen) we employ in this study. This allows for a convenient once-a-month call without the disadvantages of the need for a repeated weekly administration of the conventional build-up regimen of subcutaneous immunotherapy or the daily administration of a sublingual regimen. High treatment adherence speaks in favour of this treatment modality, given that this was an observational study with a much less compliance-compelling environment than interventional trials.

This study has clear limitations, including its observational nature and lack of a comparative group; thus, despite the promising results, we cannot establish a cause–effect relationship under this design, which requires further fit-for-purpose randomized clinical trials. These are already in course (EudraCT: 2018-003427-11). Participants were not followed after the conclusion of this study. In consequence, the long-term effect (persistence) of immunotherapy could not be evaluated. Some subgroups are quite small, which limits the available statistical power (formal power calculations for the primary endpoint are available on request). Nevertheless, subgroup-specific estimates and inferences are obtained through statistical adjustment, which is much more efficient than stratification. On the other hand, this study also has strengths, since it provides much-needed evidence for the paediatric population (especially for those <6 years of age) and successfully uses the CSMS, as recommended by the EMA and EAACI. Although observational studies lack the internal validity of clinical trials, they provide complementary valuable data with real-world relevance (external validity) [48], and thus have a place in clinical research.

In conclusion, Beltavac^®^ immunotherapy with polymerized HDM extract administered according to a rush schedule for one year seems to be a suitable modality of immunotherapy, in terms of safety, effectiveness, and convenience, for HDM-sensitized children with persistent allergic rhinitis with or without asthma. However, a larger cohort should be studied to confirm the safety and effectiveness of this immunotherapy. Importantly, the results suggest that suitability could be extrapolated to children aged under 5 years. This warrants future clinical trials in which the persistence of clinical tolerance after the cessation of immunotherapy and suitable biomarkers of response should also be addressed.

## Figures and Tables

**Table 1:** Patients’ baseline characteristics.

	With Asthma (N = 68)	Without Asthma (N = 19)	Total (N = 87)
Age, years [mean (SD)]	8.1 (2.4)	8.5 (2.1)	8.2 (2.3)
Male gender [n (%)]	43 (62.2)	14 (73.7)	57 (65.5)
Under 6 years of age [n (%)]	10 (14.7)	1 (5.3)	11 (12.6)
Male gender [n (%)]	43 (62.2)	14 (73.7)	57 (65.5)
BMI, kg/m2 [mean (SD)]	17.6 (3.0)	18.9 (4.4)	17.9 (3.4)
Presence of rhinitis [n (%)]	66 (97.1)	19 (100.0)	85 (97.7)
Presence of conjunctivitis [n (%)]	47 (69.1)	12 (63.2)	59 (67.8)
Severity of asthma [n (%)]			
Mild sporadic	26 (38.2)	–	26 (29.9)
Mild persistent	29 (42.7)	–	29 (33.3)
Moderate	13 (19.1)	–	13 (14.9)
CSMS6 score [mean (SD)]	1.1 (1.2)	0.7 (0.9)	1.0 (1.2)
CSMS4 score [mean (SD)]	1.2 (1.3)	1.0 (1.0)	1.1 (1.2)
Proportion of SFD [mean (SD)]	38.4 (39.1)	30.2 (31.6)	36.7 (37.7)
Health status VAS score [mean (SD)]	42.6 (19.6)	37.1 (14.2)	41.3 (18.6)
ASMS score [mean (SD)]	1.1 (1.2)	–	–
Prior asthma exacerbations [n (%)]	26 (38.2)	–	26 (29.9)

ASMS: combined asthma symptoms and daily medication score; CSMS4: combined (rhinitis) symptoms and medication score based only on the four nasal daily symptoms score and daily medication score; CSMS6: full combined (rhinoconjunctivitis) symptoms and medication score; SDF: symptom-free days.

**Table 2:** CSMS-adjusted mean changes (baseline–final).

	LSM (95% CI)	*p*-Value
CSMS6 (points) ^a^		
All (total score)	0.50 (0.24–0.75)	<0.001
With asthma (symptoms score)	0.24 (0.07–0.39)	0.002
Without asthma (symptoms score)	0.51 (−0.03–1.04)	0.063
With asthma (medication score)	0.11 (0.01–0.21)	0.031
Without asthma (medication score)	0.01 (−0.05–0.06)	0.799
≥6 years old (total score)	0.55 (0.26–0.84)	0.001
<6 years old (total score)	0.24 (−0.002–0.48)	0.052
≥6 years old vs. <6 years old		0.061
CSMS4 (points) ^a^		
All (total score)	0.55 (0.26–0.84)	<0.001
With asthma (symptoms score)	0.27 (0.09–0.46)	0.004
Without asthma (symptoms score)	0.62 (−0.10–1.34)	0.091
With asthma (medication score) ^c^	0.11 (0.01–0.21)	0.031
Without asthma (medication score) ^c^	0.01 (−0.05–0.06)	0.799
≥6 years old (total score)	0.61 (0.27–0.95)	0.001
<6 years old (total score)	0.26 (−0.02–0.54)	0.070
≥6 years old vs. <6 years old		0.074
Proportion of SFD (%) ^a^		
All	−38.73 (−72.91–−4.66)	0.026
With asthma	−36.81 (−75.85–2.22)	0.064
Without asthma	−44.94 (−115.52–25.64)	0.209
VAS on the perceived health status (points)^a^		
All	−36.63 (−46.66–−26.59)	<0.001
With asthma	−34.26 (−45.25–−23.27)	<0.001
Without asthma	−45.85 (−70.14–−21.57)	<0.001
ASMS (points) ^b^		
Total score	0.17 (−0.01–0.34)	0.058
Symptoms score	0.04 (−0.001–0.09)	0.058
Medication score	0.03 (−0.02–0.07)	0.224
Incidence of asthma exacerbations ^b^		
Change with respect to the previous year (relative risk)	0.31 (0.18–0.54)	<0.001

95% CI: 95% confidence interval; ASMS: combined asthma symptoms and daily medication score; CSMS4: combined (rhinitis) symptoms and medication score based only on the four nasal daily symptoms score and daily medication score; CSMS6: full combined (rhinoconjunctivitis) symptoms and medication score; LSM: least square means; SDF: symptom-free days. ^a^ Results estimated for the subgroups of interest [patients with (n = 68) and without asthma (n = 19), patients with (n = 59) and without rhino-conjunctivitis (n = 28), or patients aged ≥6 years old (n = 76) and <6 years old (n = 11)] from the regression models using the whole population. ^b^ Only for the subgroup of children with asthma. ^c^ These rows are redundant between CSMS6 and CSMS4 scales.

**Table 3:** Accumulated incidences of adverse reactions and adverse events.

	n (%)	90% CI of %
Adverse reactions		
At least one	8 (9.20)	4.10–14.29
At least one serious	0 (0.0)	0.00–0.001
At least one local	8 (9.20)	4.10–14.29
At least one local major	3 (3.45)	0.23–6.67
At least one local minor	5 (5.75)	1.64–9.85
At least one systemic	2 (2.30)	0.00–4.94
At least one systemic grade 1	1 (1.15)	0.00–3.03
At least one systemic grade 2	1 (1.15)	0.00–3.03
At least one systemic grade 3	0 (0.0)	0.00–0.001
At least one systemic grade 4	0 (0.0)	0.00–0.001
At least one immediate	1 (1.15)	0.00–3.03
At least one delayed	8 (9.20)	4.10–14.29
At least one with the initial dose of 0.2 mL	3 (3.45)	0.23–6.67
At least one with the initial dose of 0.3 mL	5 (5.75)	1.64–9.85
At least one requiring treatment	6 (6.90)	2.43–11.37
Adverse events		
At least one	23 (26.44)	18.66–34.21
At least one serious	3 (3.45)	0.23–6.67
At least one requiring treatment	17 (19.54)	12.55–26.53

## Data Availability

Dr. Alfonso Malet I. Casajuana, from the Department of Allergy of Teknon Hospital, Barcelona (Spain), and Dr. Inma Buendia Jimenez from the Medical Affairs Department of Probelte Pharma will oversee the dataset. Granting access to this information will be evaluated on a case-by-case basis, upon reasonable request by the interested party. Data access requests should be addressed to Inma Buendia at inmaculadabuendia@probeltepharma.es.

## Supplementary Materials

The following supporting information can be downloaded at: https://www.mdpi.com/article/10.3390/jcm14124188/s1, Table S1: CSMS individual symptom-adjusted mean changes (baseline–final). Table S2: Specific Ig-adjusted mean changes (baseline–final). Figure S1: Evolution of serum-specific IgE. Figure S2: Evolution of serum-specific IgG4.
