# Supplementary material for: Subcutaneous House Dust Mite Immunotherapy Effectiveness and Safety in a Paediatric Population: A Prospective Real-Life Study
Source: J Clin Med. 2025 Jun 12;14(12):4188. doi: 10.3390/jcm14124188 (PMC12193782; doi:10.3390/jcm14124188)
Supplement: Supplementary file 1 [file jcm-14-04188-s001.zip › jcm-3584274-supplementary.pdf]

Supplementary Data

Table S1: CSMS individual symptoms adjusted mean changes (Baseline – Final)

	LSM (95% CI)	p-value
**Nasal symptoms (points)**		
Itchy nose	0.22 (0.06 – 0.38)	0.006
Sneezing	0.34 (0.11 – 0.57)	0.004
Runny nose	0.31 (0.09 – 0.54)	0.007
Blocked nose	0.13 (0.03 – 0.23)	0.012
**Conjunctival symptoms (points)**		
Itchy/red eyes	0.12 (0.03 – 0.21)	0.007
Watery eyes	0.04 (0.01 – 0.08)	0.022

CSMS: full Combined Symptom and Medication Score; LSM: Least Square Means; 95% CI: 95% Confidence Interval

Table S2: Specific Ig adjusted mean changes (Baseline – Final)

	LSM (95% CI)	p-value
**IgE (kU/L)**		
Anti-Der P	−2.71 (−20.98 – 15.55)	0.768
Anti-Der f	−17.74 (−56.62 – 19.06)	0.339
Anti-Der p 1	−29.84 (−78.23 – 18.55)	0.223
Anti-Der p 2	−31.39 (−83.01 – 20.22)	0.230
Anti-Der p 10	−0.38 (−0.75 – −0.003)	0.049
Anti-Der p 23	−10.87 (−41.62 – 19.84)	0.482
**IgG_4 (kU/L)**		
Anti-Der P	−5.42 (−8.80 – −2.04)	0.002
Anti-Der f	−7.49 (−13.97 – −1.01)	0.024
Anti-Der p 1	−1.58 (−3.93 – 0.78)	0.181
Anti-Der p 2	−7.50 (−19.62 – 4.62)	0.216
Anti-Der p 10	−0.01 (−0.04 – 0.01)	0.219
Anti-Der p 23	−0.19 (−0.49 – 0.11)	0.197

CSMS: full Combined Symptom and Medication Score; LSM: Least Square Means; 95% CI: 95% Confidence Interval

Figure S1: Evolution of serum specific IgE

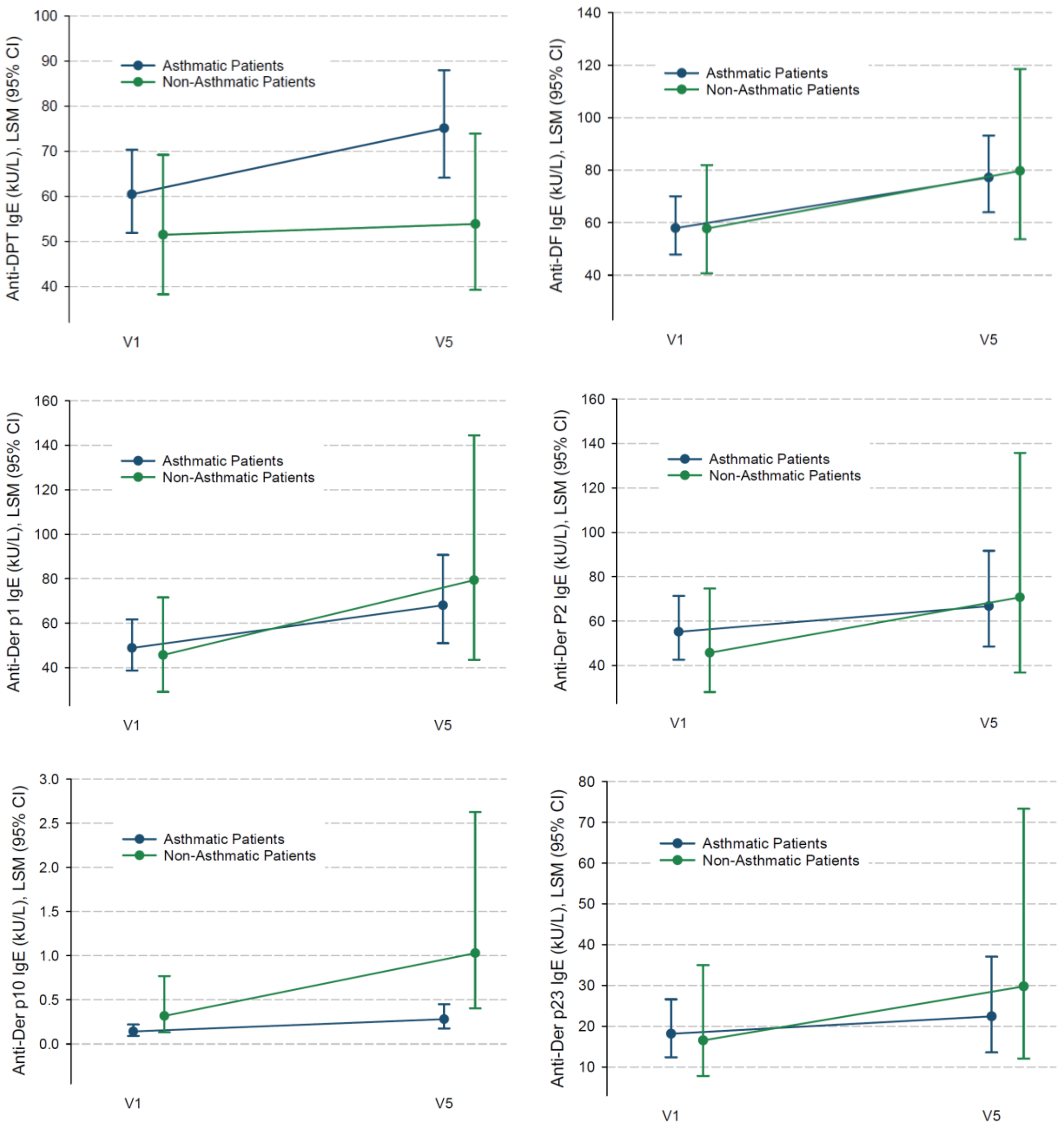

DPT: Dermatophagoides pteronyssinus; DF: Dermatophagoides farinae; IgE: Immunoglobulin E; V1: Visit 1; V5: Visit 5

Figure S2: Evolution of serum specific IgG_4

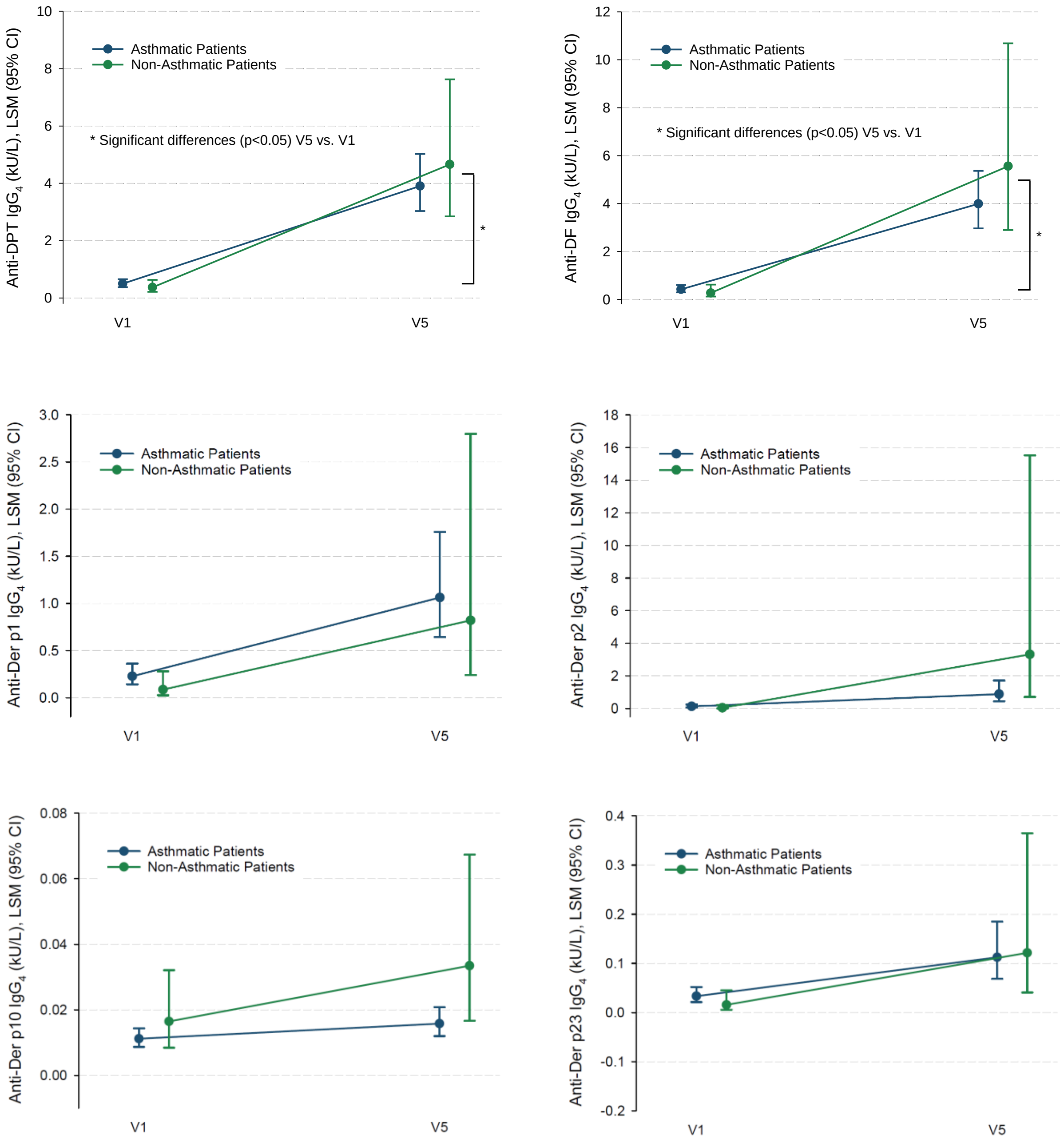

DPT: Dermatophagoides pteronyssinus; DF: Dermatophagoides farinae; IgG4: Immunoglobulin G subtype 4; V1: Visit 1; V5: Visit 5
